# Molecular and Biological Characterization of *Spodoptera frugiperda* Multiple Nucleopolyhedrovirus Field Isolate and Genotypes from China

**DOI:** 10.3390/insects11110777

**Published:** 2020-11-10

**Authors:** Chengfeng Lei, Jian Yang, Jia Wang, Jia Hu, Xiulian Sun

**Affiliations:** 1 Wuhan Institute of Virology, Center for Biosafety Mega-Science, Chinese Academy of Sciences, Wuhan 430071, China; cflei@wh.iov.cn (C.L.); jemappelleyangjian@zju.edu.cn (J.Y.); wangjia_whiov@163.com (J.W.); hujia@wh.iov.cn (J.H.); 2 College of Life Science, University of Chinese Academy of Sciences, Beijing 100049, China

**Keywords:** SfMNPV, field isolate, genotype, molecular characterization, biological activity, OB yield

## Abstract

**Simple Summary:**

The fall armyworm is a notorious lepidopteran pest that consumes many economically important crops. Its recent invasion into China threatens crops in over 19 provinces. This species is susceptible to its homologous nucleopolyhedrovirus (*Spodoptera frugiperda* multiple nucleopolyhedrovirus, or SfMNPV). Selection of indigenous isolates that are adaptable in each geographical region is important for developing a virus-based pesticide. In this study, an SfMNPV field isolate was obtained from a natural population of the fall armyworm in Hubei, China. Two genotypes were cloned from the field isolate, and one genotype, SfHub-A, which had similar activity to the field isolate and produced significantly more progeny viruses, was considered to be a suitable strain for the commercial production of SfMNPV. This information will be valuable for developing a virus-based pesticide against fall armyworm in China.

**Abstract:**

The fall armyworm, *Spodoptera frugiperda*, is a new invading pest in China. The baculovirus *Spodoptera frugiperda* multiple nucleopolyhedrovirus (SfMNPV) is a pathogenic agent of the fall armyworm and a potential agent for its control in integrated pest management strategies. In this work, we analyze the molecular and biological characteristics of an SfMNPV isolate collected from maize in China (SfMNPV-Hub). Two genotypes were further isolated from SfMNPV-Hub by an in vivo cloning method. The PstI profile of one genotype (SfHub-A) was similar to genotype A of the SfMNPV Colombian isolate, and the other (SfHub-E) was similar to genotype E of the Colombian isolate. The bioactivity of SfHub-A against second-instar *S. frugiperda* larvae was not significantly different from that of SfMNPV-Hub, whereas SfHub-E was 2.7–5.5 fold less potent than SfMNPV-Hub. The speed of kill of SfHub-E was quicker than SfMNPV-Hub, while SfHub-A acted slower than SfMNPV-Hub. Occlusion body (OB) production of SfHub-A in an *S. frugiperda* cadaver was significantly higher than that of SfMNPV-Hub, while SfHub-E yielded far fewer occlusion bodies (OBs) in the host larvae. These results provide basic information for developing a virus-based pesticide against the invading pest *S. frugiperda*.

## 1. Introduction

The fall armyworm, *Spodoptera frugiperda* (Lepidoptera: Noctuidae), is a highly fecund, adaptable, and polyphagous lepidopteran that consumes many plant species, including economically important crops, such as maize, soybeans, and sorghum [1]. A serious pest in most of North and South America, it was recently introduced into Africa, Asia, and Oceania, where it now threatens crops in over 110 countries [2]. In China, *S. frugiperda* was first found in Yunnan province in 2018, from where it rapidly spread to 19 provinces, posing a serious threat to China’s agricultural production [3].

Baculoviruses are important pathogens for a wide range of insect pests [4,5]. Field observations indicate that *S. frugiperda* is susceptible to a homologous *Spodoptera frugiperda* multiple nucleopolyhedrovirus (SfMNPV) that causes natural epizootics of the disease [6]. This has motivated studies focused on the development of SfMNPV as a biological insecticide [7,8,9,10]. Although several SfMNPV isolates from the United States, Nicaragua, Brazil, Argentina, and Colombia have been biologically or genetically characterized [7,11,12,13,14,15,16,17,18,19], the selection of indigenous isolates that are suitable for development as biological control agents requires the characterization of those isolates present in each geographical region [20]. In Mexico, laboratory and field studies have determined that indigenous isolates of nucleopolyhedrovirus (NPV) have the potential for use in the biological control of *S. frugiperda* [8,9,21].

Here, we describe the isolation an SfMNPV from a natural population of *S. frugiperda* from maize crops in Hubei Province, China, which we designate as *Spodoptera frugiperda* multiple nucleopolyhedrovirus Hub (SfMNPV-Hub). We further cloned two genotypes from the SfMNPV field isolate, and their molecular and biological characteristics were compared with the field isolate.

## 2. Materials and Methods

### 2.1. Insects, Virus Isolation, and Purification

A population of *S. frugiperda* larvae were collected from maize crops in Yuanjiapu Village, Huangzhou District (30°43′ N, 114°88′ E), Huanggang City, Hubei Province, China, in June 2019. The larvae were reared on lettuce leaves in a round plastic cup with 200 mL of volume, covered with a plastic cover with tiny pores and lined with soft tissue (Vinda, Jiangmen, China). The second and further generations of *S. frugiperda* were reared on an artificial diet [22] and kept in an acclimatized room at 27 ± 1 °C, 65% ± 5% relative humidity, and a 16:8 (light/dark) photoperiod. The eggs and pupae of each generation were disinfected with 4% formaldehyde for 15 min, rinsed with distilled H_2_O three times, and dried naturally.

SfMNPV-Hub was obtained from a *S. frugiperda* cadaver with characteristics of baculovirus infection in the first rearing round in the laboratory. The cadaver was homogenized with 1 mL of 1× PBS buffer (Phosphate buffered saline buffer) (137 mmol/L NaCl, 2.7 mmol/L KCl, 10 mmol/L Na_2_HPO_4_, 2 mmol/L KH_2_PO_4_, pH 7.4), filtered on cotton gauze, and centrifuged at 5000× *g* for 30 min. The pellet was suspended in 1× PBS buffer and centrifuged at 100× *g* for 5 min. The process was performed two more times. The pellet was suspended in 1× PBS buffer. Occlusion bodies (OBs) were stored at 4 °C and used to inoculate the laboratory reared larvae of *S. frugiperda* to confirm virus etiology and propagate OBs.

### 2.2. Restriction Endonuclease (REN) Analyses, PCR Amplification, and Sequencing Analyses

Virus DNA samples were purified from OBs, as previously described [23], and digested with PstI and BamHI (Takara Bio, Inc., Otsu, Japan). Restriction endonuclease (REN) profiles were visually analyzed using the Syngene G: BOX Chemi imaging system (Syngene, Cambridge, United Kingdom). A DNA marker (Trans Gene Biotech, Beijing, China) was used to indicate the molecular size of the DNA fragments.

The amplification of partial *polh*, *lef-8*, and *lef-9* genes were performed using degenerate baculoviruses primer pairs (prPH-1, prPH-2, prL8-1, prL8-2, prL9-1, and prL9-2) and same reaction conditions as mentioned in Jehle et al. [24]. The PCR products were purified by using the Cycle-Pure Kit (OMEGA bio-tek, Norcross, United States), and were sequenced by automatic sequencing (Sangon Biotech, Shanghai, China). Partial *polh*, *lef-8*, and *lef-9* sequences were submitted to the GenBank with the accession numbers MW115954, MW115952, and MW115953. The Kimura 2-parameter (K-2-P) distances of *lef-8*, *lef-9*, and *polh* fragments among SfMNPV-Hub and other seven sequenced SfMNPV isolates were calculated using Kimura 2-parameter model by MEGA 7.0 [25].

### 2.3. In Vivo Cloning Procedure

A modification of the method described by Smith and Crook [26] was used to in vivo clone the genotypes in the field isolate. Fourth-instar *S. frugiperda* larvae were dosed with a dilution series of SfMNPV-Hub (1 × 10^7^, 1 × 10^6^, 1 × 10^5^, 1 × 10^4^, and 1 × 10^3^ OBs/mL), and the 5% lethal concentration (LC_5_) was calculated by probit analysis using the “drc” package in R (version 3.6.1) [27]. OBs at LC_5_ were used to further inoculate the fourth-instar larvae, and the proliferated OBs were collected and purified from individual cadavers. DNA was isolated and subjected to REN analyses. The isolates with REN profiles differing from those of SfMNPV-Hub were thought to be potential new genotypes, and were subjected to two more rounds of the in vivo cloning procedure. 

### 2.4. Scanning Electron Microscopy (SEM) and Transmission Electron Microscopy (TEM)

For scanning electron microscopy (SEM), purified OBs were spread onto a piece of foil paper, dried naturally overnight, sputter-coated with gold, and observed with a scanning electron microscope (SU8010, Hitachi, Tokyo, Japan) at an accelerating voltage of 10 kV. The diameters of OBs of the field isolate and genotypes were measured and compared by the Tukey’s honestly significant difference (HSD) test, followed by analysis of variance (ANOVA) using R (version 3.6.1).

For transmission electron microscopy (TEM), the purified OBs were resin-embedded, sectioned, and stained with lead citrate for 30 min, as described previously [28]. Stained ultrathin sections were examined with a transmission electron microscope (H-800, Hitachi, Tokyo, Japan) at an accelerating voltage of 200 kV.

### 2.5. Bioassay

The infectivity of the SfMNPV field isolate and genotypes were measured by the droplet-feeding method. Second-instar larvae were fed OBs diluted into five concentrations (1.92 × 10^3^, 9.6 × 10^3^, 4.8 × 10^4^, 2.4 × 10^5^, and 1.2 × 10^6^ OBs/mL). After fasting overnight, larvae were fed a mixture of 4% sucrose, sufficient blue dye erioglaucine (Aladdin Industrial Corporation, Shanghai, China), and different concentrations of OBs for 10 min. Larvae with blue guts were transferred to 24-well plates containing the artificial diet. Forty-eight insects were tested per concentration in each assay. The larvae were incubated under the same conditions as the healthy larvae mentioned above. Mortality was recorded daily till all of the larvae had either pupated or died. Bioassays were performed in duplicate. The median lethal concentration (LC_50_) values and potency ratio of the genotypes to the field isolate were calculated using the “drc” package in R (version 3.6.1) [27].

To determine the killing speed of the SfMNPV variants, second-instar larvae were fed OBs at 100 × LC_50_, using the droplet-feeding method mentioned above. The larvae were checked for mortality approximately every 8 h over the initial 56 h post-inoculation (h.p.i.), every 4 h (during daylight) or 8 h (during night) between 56 h.p.i. and 100 h.p.i., and every 12 h till all larvae had either pupated or died. Median survival times (ST_50_) and their 95% confidence intervals (CIs) were calculated using Kaplan–Meier curves with the “survival” package in R (version 3.6.1). Differences between survival curves of the test larvae dosed with the SfMNPV variants were compared using the log–rank test [29].

### 2.6. OB Production

To determine the OB production in an individual cadaver, fourth-instar larvae were exposed to 1 × 10^7^ OBs/mL by the droplet method mentioned above. Larvae that had ingested the OB suspension were transferred to a fresh diet and reared further individually, under the same conditions mentioned above. Individuals showing terminal disease symptoms of baculovirus infection (discoloration, no movement) were transferred to microtubes prior to death and maintained at room temperature. Larvae were liquefied and immediately stored at −20 °C. To measure OB production, individual cadavers were sonicated for 2 min, 1× PBS buffer was added to a total volume of 1 mL, and the mixture was homogenized on a vortexer. OBs in the homogenate were counted using a hemocytometer after appropriate dilution. OB production per cadaver of SfMNPV variants was compared with the field isolate by Dunnett’s multiple comparisons test, followed by ANOVA using R (version 3.6.1).

## 3. Results

### 3.1. Virus Isolation and TEM 

**Figure 1:**
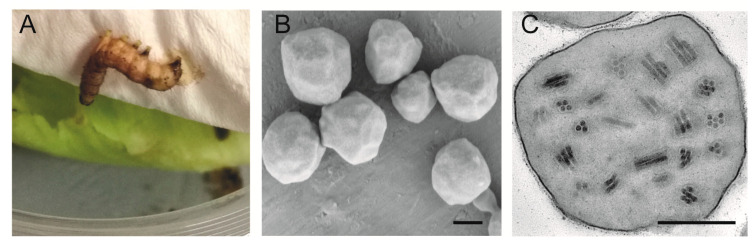
Image of *Spodoptera frugiperda* larval cadaver showing symptoms of baculovirus infection when reared with the maize crop *S. frugiperda* larvae in laboratory (**A**), and scanning electron microscopy (SEM) (**B**) and transmission electron microscopy (TEM) (**C**) observation of the isolated *Spodoptera frugiperda* multiple nucleopolyhedrovirus (SfMNPV) occlusion bodies (OBs). Scale bars = 0.5 μm.

When we reared *S. frugiperda* larvae from maize on lettuce leaves in laboratory, one cadaver showed typical characteristics of baculovirus infection, i.e., hanging under the tissue underlaid the cover of the rearing cup, body swollen with a pus-like discharge (Figure 1A). The OBs were isolated and inoculated to fourth-instar larvae reared on the artificial diet. The larvae showed typical symptoms of baculovirus infection, and numerous OBs could be collected from the cadavers. SEM and TEM revealed the OBs to be polyhedral or irregular in shape (Figure 1B), containing many enveloped virions packaged with either single or multiple nucleocapsids within an envelope (Figure 1C). Thus, the virus isolated from *S. frugiperda* larvae collected in Hubei Province, China, might be an alphabaculovirus, and was designated SfMNPV-Hub.

### 3.2. In Vivo Cloning of SfMNPV Genotypes

**Figure 2:**
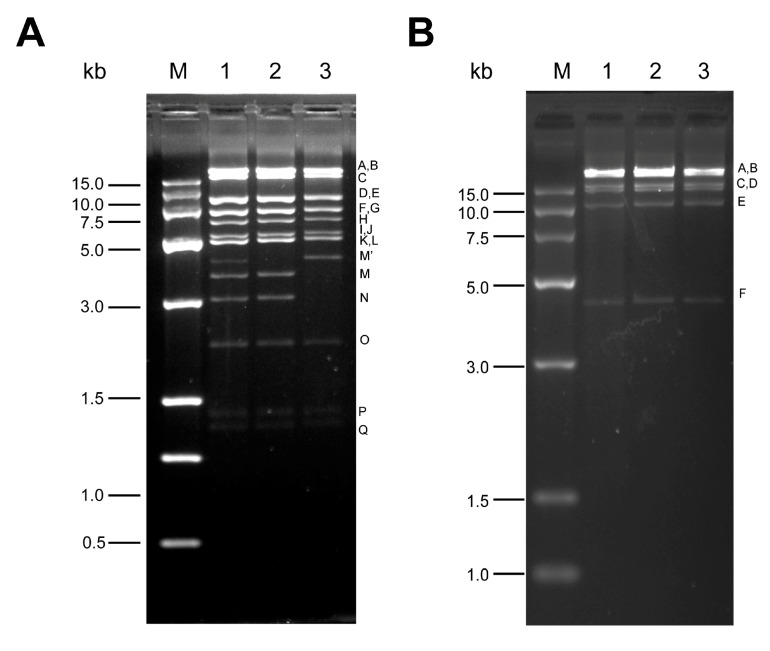
Restriction endonuclease (REN) profiles of a SfMNPV field isolate and genotypes digested with PstI (**A**) and BamHI (**B**), generated by electrophoresis on a 1% agarose gel. Lane 1: SfMNPV-Hub, Lane 2: SfMNPV-Hub (SfHub)-A, Lane 3: SfHub-E. All DNA segments were marked with a letter corresponding to their sizes. M: DNA marker, with the size of each segment was shown on the left.

When fourth-instar larvae were exposed to a dilution series of SfMNPV-Hub, the LC_5_ value was 1.3 × 10^3^ OBs/mL. In total, 4520 fourth-instar larvae were dosed with SfMNPV-Hub OBs at LC_5_, of which 203 died of viral infection, resulting in a mortality rate of 4.5%. DNA samples were isolated from OBs of each cadaver and digested with REN. Both the PstI and BamHI profiles of SfMNPV-Hub were similar to those of SfMNPV Colombian isolate [17], while there was a submolecular band between the L and M fragments, indicated as M’, of the PstI profile (Figure 2, lane 1), indicating that the SfMNPV-Hub field isolate consisted of several genotypes. When SfMNPV-Hub was subjected to in vivo cloning, two genotypes were found (Figure 2A); the PstI profile of one genotype was similar to genotype A of the SfMNPV Colombian isolate, and the other was similar to genotype E of the Colombian isolate [19]. 

All Kimura 2-parameter distances between the partial *polh*, *lef-8*, and *lef-9* sequences of SfMNPV-Hub and the other SfMNPV isolate were smaller than 0.015 (Supplementary Materials, Table S1). Thus, we designated the two genotypes SfHub-A and SfHub-E, respectively. 

**Figure 3:**
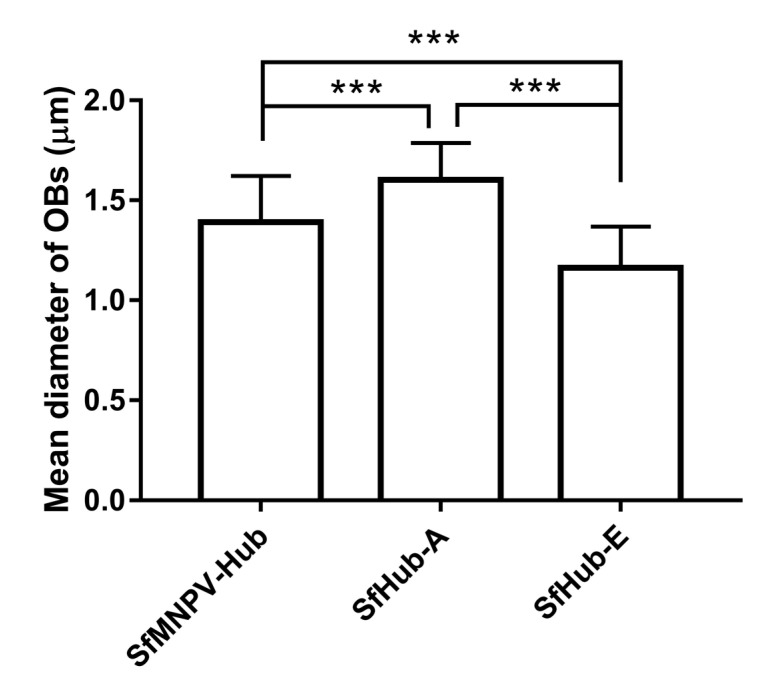
Diameter of OBs of SfMNPV-Hub (*n* = 128), SfHub-A (*n* = 111), and SfHub-E (*n* = 175). Error bars represent the standard deviation (SD). *** represents *p* < 0.001 by Tukey’s honestly significant difference (HSD).

OB diameters of the SfMNPV field isolate and genotypes were measured by SEM. OB diameters of SfHub-A (1.602 ± 0.184 μm) were significantly larger than those of SfMNPV-Hub (1.390 ± 0.232 μm), while SfHub-E OBs (1.132 ± 0.206 μm) were significantly smaller (Figure 3).

**Figure 4:**
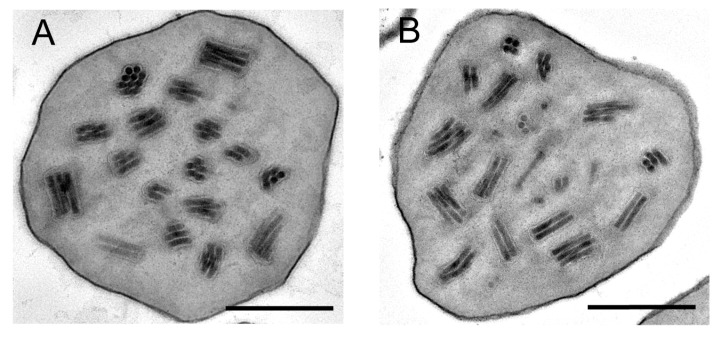
TEM micrograph of sectioned OBs of SfHub-A (**A**) and SfHub-E (**B**). Scale bars = 0.5 μm.

TEM observation showed that the OBs of the SfMNPV genotypes contained normal occlusion-derived virions (ODVs) and were similar to those of SfMNPV-Hub (Figure 4). The number of nucleocapsids enveloped in each ODV ranged from 1 to 10.

### 3.3. Biological Activity of SfMNPV Field Isolate and Genotypes

The infectivity of the SfMNPV field isolates and genotypes was assayed in second-instar larvae. In both test 1 and 2, the LC_50_ of SfHub-A did not significantly differ from that of SfMNPV-Hub, whereas the LC_50_ of SfHub-E was 2.7–5.5-fold higher than that of SfMNPV-Hub (Table 1). 

When second-instar larvae were inoculated at the concentration of 100 × LC_50_, the ST_50_ of SfHub-A was significantly longer than that of SfMNPV-Hub (Table 2), while the ST_50_ of SfHub-E was significantly shorter (Table 2). This result indicates that SfHub-E killed the larvae quicker than SfMNPV-Hub, while SfHub-A acted slower than SfMNPV-Hub.

### 3.4. Production of SfMNPV Field Isolate and Genotypes In Vivo

**Figure 5:**
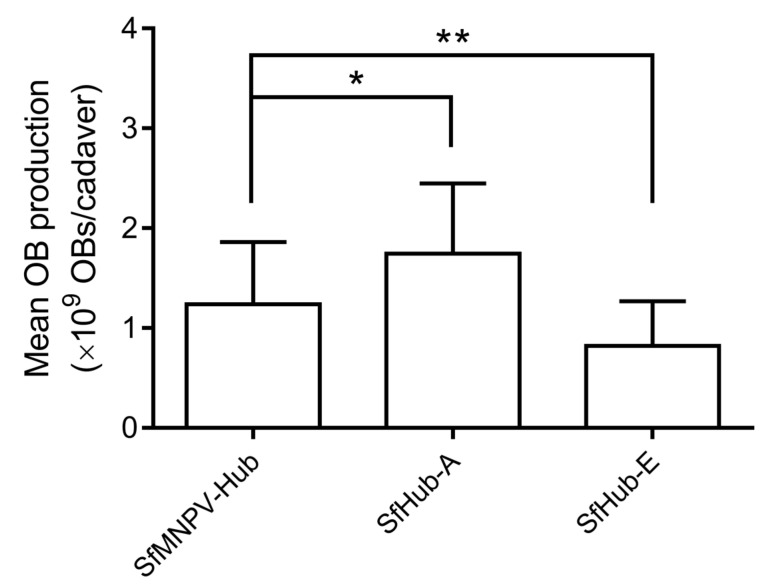
OB production of SfMNPV field isolate and genotypes in the *S. frugiperda* larvae. OB numbers in 39, 25, and 37 cadavers killed by SfMNPV-Hub, SfHub-A, and SfHub-E were counted. Error bars represent the SD. * represents *p* < 0.05, ** represents *p* < 0.01 by Dunnett’s test.

When fourth-instar larvae were inoculated with the field isolate and genotypes, SfHub-A yielded 1.745 ± 0.705 × 10^9^ OBs/cadaver, which was significantly higher than SfMNPV-Hub (1.240 ± 0.621 × 10^9^ OBs/cadaver) (*q* = 3.363, *p* = 0.0022), whereas SfHub-E yielded significantly fewer OBs (0.821 ± 0.445 × 10^9^ OBs/cadaver) than SfMNPV-Hub (*q* = 3.107, *p* = 0.0048) (Figure 5).

## 4. Discussion

China is the largest producer and consumer of pesticides [30]. To control the proliferation of *S. frugiperda*, pesticides that are safe, effective, and compatible with the environment are required. Baculoviruses have been developed as commercial bioinsecticides, because they are easily compatible with integrated pest management strategies [31]. SfMNPV has been developed as a commercial insecticide [32], and the selection of indigenous isolates is important for developing a virus-based pesticide against *S. frugiperda* [20].

Field isolates of baculoviruses rarely contain a single genotype, and variants can be separated by in vitro or in vivo cloning techniques [33]. SfMNPV isolated from different geographic regions frequently shows differences in REN profiles [7,34]. Ten genotypic variants have been isolated from the SfMNPV Colombian field isolate by in vitro virus cloning, named SfCOL-A, through J [18]. Using a similar method, nine distinct genotypes were identified from an SfMNPV Nicaraguan (SfNIC) isolate [13]. The in vivo cloning technique is based on the hypothesis that larvae inoculated at a very low concentration of virus (such as LC_5_) will result in the replication of a single infectious particle, and progeny viruses are clones of a single genotype [26]. This hypothesis has been proven by successfully isolating single genotypes from several wild type baculoviruses, including granuloviruses (GVs) [26,35] and NPVs [26,36,37]. For GVs and single nucleocapsid NPVs, three rounds of in vivo cloning are typically sufficient to produce pure genotypes [26,35], while for the multi-capsid NPVs (MNPV), more rounds are usually required. For example, some genotypes of *Spodoptera exigua* MNPV needed up to six passages to be purified [36]. In the present study, we successfully purified two genotypes of the MNPV from *S. frugiperda* by three rounds of in vivo cloning. We checked more than 200 individual cadavers by REN, yielding only two genotypes. The low number of genotypes may be due to the fact that SfMNPV-Hub was isolated from a single host insect, which could have limited virus diversity in the isolate.

Biological activity of different genotypes of the same virus can differ largely. OBs of all genotypic variants cloned from the SfNIC isolate are less pathogenic than the wild-type OBs or those of the experimental mixtures of genotypes [12,13,38]. In a commercial isolate of *S. exigua* MNPV, genotypes with the deletion of several genes showed lower activity than the field population [36,39]. In our study, activity of SfHub-A did not significantly differ from that of the field isolate, while SfHub-E was significantly less virulent. This may be because SfHub-E lacks the genes *gp37*, *ptp-2*, *egt*, *sf27*, *sf28*, and *sf29* [19]. It has been reported that chitin-binding protein GP37 has a synergistic effect on the infectivity of NPVs [40]. Other genes missing in SfHub-E may also be related to virulence. The diameter of SfHub-E OBs was significantly smaller than that of SfHub-A, possibly due to the lack one of the aforementioned genes.

In this study, the speed of kill of SfHub-E was quicker than SfHub-A, which could be a consequence of the lack of the *egt* gene, which has been frequently reported to affect the killing speed of baculoviruses [16,23,41,42,43]. The ST_50_ values of the SfMNPV field isolate and genotypes against second-instar larvae were between 62.5–95.0 h, which is much shorter than those of the SfNIC isolates (90–140 h) [13] and SfCOL genotypic variants (124–178 h) in the second-instar larvae [18].

OB production of different genotypes of the same virus may also vary. OB production is higher in the SfCOL-wt isolate than in any of the component genotypes, or mixtures thereof [18]. OB production of SfMNPV 3AP2-inoculated larvae was significantly lower than that in SfMNPV Sf3-inoculated larvae [44]. When inoculated using SfNIC in the second-instar, the most productive infections are those of genotype A and A + C, followed by the SfNIC field isolate [45]. Compared to the SfNIC field isolate, OB production is greatly reduced in insects infected by genotype B, presumably due to the rapid speed of kill of this genotype. In our study, OB production of SfHub-E was significantly lower than that of SfMNPV-Hub and SfHub-A.

Several characteristics of a baculovirus isolate or genotype, i.e., bioactivity, killing speed, and production, are considered when evaluating suitability for use as a commercial, virus-based insecticide. From our experiments, SfHub-A had similar activity as the SfMNPV-Hub field isolate and produced significantly more OBs, but required 11–18 h longer to kill the host larvae. In a modeling study, the killing speed of a baculovirus does not contribute to its control efficacy as much as its activity [31]. Considering the significantly higher OB production, we believe SfHub-A to be a suitable strain for the commercial production of SfMNPV in China.

## 5. Conclusions

An SfMNPV field isolate was obtained from a natural population of the newly invading pest *S. frugiperda* in China. Two genotypes were cloned from the field isolate, and one genotype, SfHub-A, which had similar activity to the field isolate and produced significantly more OBs, was considered to be a suitable strain for the commercial production of SfMNPV. This information will be valuable for developing a virus-based pesticide against *S. frugiperda* in China.

## Figures and Tables

**Table 1:** Median lethal concentrations (LC_50_) of SfMNPV field isolate and genotypes against second-instar *Spodoptera frugiperda* larvae.

Test	Virus	LC_50_ (95% CI) (× 10^5^ OBs/mL)	*χ*^2^/d.f.	Potency Ratio (95% CI) to SfMNPV-Hub ^1^
1	SfMNPV-Hub	1.68 (0.59, 8.23)	5.768/3	-
	SfHub-A	2.36 (0.72, 16.46)	6.275/3	0.710 (0.343, 1.472)
	SfHub-E	9.28 (4.80, 25.52)	0.694/3	0.181 (0.071, 0.460) *
2	SfMNPV-Hub	1.35 (0.33, 13.84)	8.668/3	-
	SfHub-A	1.27 (0.38, 7.10)	5.071/3	1.059 (0.488, 2.298)
	SfHub-E	3.66 (1.23, 28.85)	5.530/3	0.368 (0.171, 0.789) *

^1^ The potency ratio was calculated by dividing the LC_50_ of SfMNPV-Hub by those of the genotypes. * indicated that the potency of the genotype was significantly different from that of SfMNPV-Hub based on whether the 95% CI of the potency ratio contained 1.0 [28]. OB: occlusion body; d.f.: degrees of freedom.

**Table 2:** Median survival times (ST_50_) of the second-instar *S. frugiperda* larvae inoculated with the SfMNPV field isolate and genotypes.

Test	Virus	*n*	ST_50_ (95% CI) (h.p.i.) ^1^	*χ* ^2^	*p*
1	SfMNPV-Hub	49	84.0 (79.7, 88.3)		
	SfHub-A	50	95.0 (91.5, 98.5)	11.211	0.001
	SfHub-E	48	72.0 (69.5, 79.5)	11.118	0.001
2	SfMNPV-Hub	45	77.0 (71.2, 82.8)		
	SfHub-A	51	95.0 (92.6, 97.4)	21.817	<0.001
	SfHub-E	48	62.5 (52.4, 72.6)	8.827	0.003

^1^ ST_50_ values of insects were determined by the Kaplan–Meier estimator and are reported with 95% CIs. h.p.i.: hours post inoculation; d.f.: degrees of freedom.

## Supplementary Materials

The following are available online at https://www.mdpi.com/2075-4450/11/11/777/s1, Table S1: The pairwise Kimura 2-parameter distances of the nucleotide sequences of (A) *polh*, (B) *lef-8*, and (C) *lef-9* fragments among Spodoptera frugiperda multiple nucleopolyhedrovirus (SfMNPV) isolates.
