# Supplementary material for: Molecular and Biological Characterization of Spodoptera frugiperda Multiple Nucleopolyhedrovirus Field Isolate and Genotypes from China
Source: Insects. 2020 Nov 10;11(11):777. doi: 10.3390/insects11110777 (PMC7697700; doi:10.3390/insects11110777)
Supplement: Supplementary file 1 [file insects-11-00777-s001.pdf]

Supplementary

Table 1. The pairwise K-2-P distances of the nucleotide sequences of (A) *polh,* (B)*lef-8*, and (C)*lef-9* fragments among Spodoptera frugiperda multiple nucleopolyhedrovirus (SfMNPV) isolates.

A:

polh **(γ = 1.80 [1])**	**1**	**2**	**3**	**4**	**5**	**6**	**7**	**8**	**9**
1 SfMNPV-Hub									
2 SfMNPV_G	0.000								
3 SfMNPV_Ni	0.000	0.000							
4 SfMNPV_IIB1197	0.000	0.000	0.000						
5 SfMNPV_19	0.000	0.000	0.000	0.000					
6 SfMNPV_IIB459	0.000	0.000	0.000	0.000	0.000				
7 SfMNPV_Co	0.002	0.002	0.002	0.002	0.002	0.002			
8 SfMNPV_3AP2	0.002	0.002	0.002	0.002	0.002	0.002	0.004		
9 MabrMNPV_K1 [2]	0.208	0.208	0.208	0.208	0.208	0.208	0.205	0.205	

B:

lef-8 **(γ= 0.64 [1])**	**1**	**2**	**3**	**4**	**5**	**6**	**7**	**8**	**9**
1 SfMNPV-Hub									
2 SfMNPV_G	0.003								
3 SfMNPV_Ni	0.003	0.000							
4 SfMNPV_IIB1197	0.003	0.000	0.000						
5 SfMNPV_19	0.004	0.004	0.004	0.004					
6 SfMNPV_IIB459	0.003	0.003	0.003	0.003	0.004				
7 SfMNPV_Co	0.001	0.001	0.001	0.001	0.003	0.001			
8 SfMNPV_3AP2	0.013	0.010	0.010	0.010	0.012	0.013	0.012		
9 MabrMNPV_K1 [2]	0.843	0.843	0.843	0.843	0.843	0.833	0.833	0.859	

C:

lef-9 **(γ = 200 [1])**	**1**	**2**	**3**	**4**	**5**	**6**	**7**	**8**	**9**
1 SfMNPV-Hub									
2 SfMNPV_G	0.007								
3 SfMNPV_Ni	0.007	0.000							
4 SfMNPV_IIB1197	0.007	0.000	0.000						
5 SfMNPV_19	0.007	0.000	0.000	0.000					
6 SfMNPV_IIB459	0.007	0.000	0.000	0.000	0.000				
7 SfMNPV_Co	0.007	0.000	0.000	0.000	0.000	0.000			
8 SfMNPV_3AP2	0.007	0.000	0.000	0.000	0.000	0.000	0.000		
9 MabrMNPV_K1 [2]	0.232	0.227	0.227	0.227	0.227	0.227	0.227	0.227	

[1] The gamma shape parameter was calculated for each alignment with MEGA v7.0 [1]. 2 Mamestra brassicae multiple nucleopolyhedrovirus (MabrNPV) K1 isolate (GenBank: JQ798165.1) [2] was used as an outgroup control.

References

1. Kumar, S.; Tamura, K.; Nei, M. MEGA3: Integrated software for molecular evolutionary genetics analysis and sequence alignment. Briefings in Bioinformatics 2004, 5, 150-163.
2. Choi,J.B.; Heo,W.I.; Shin,T.Y.; Bae,S.M.; Kim,W.J.; Kim,J.I.; Kwon,M.; Choi,J.Y.; Je,Y.H.; Jin,B.R.; and Woo,S.D. Complete genomic sequences and comparative analysis of Mamestra brassicae nucleopolyhedrovirus isolated in Korea. Virus Genes 2013, 47(1), 133-151.
